# Neuropilin1 Expression Acts as a Prognostic Marker in Stomach Adenocarcinoma by Predicting the Infiltration of Treg Cells and M2 Macrophages

**DOI:** 10.3390/jcm9051430

**Published:** 2020-05-12

**Authors:** Ji Young Kang, Minchan Gil, Kyung Eun Kim

**Affiliations:** 1 Department of Health Industry, Sookmyung Women’s University, Seoul 04310, Korea; ellykang@sookmyung.ac.kr; 2 Department of Stem Cell and Regenerative Biotechnology, Konkuk University, Seoul 05029, Korea; minchangil@gmail.com; 3 Department of Cosmetic Sciences, Sookmyung Women’s University, Seoul 04310, Korea; 4 Nano-Bio Resources Center, Sookmyung Women’s University, Seoul 04310, Korea

**Keywords:** neuropilin1 (NRP1), stomach adenocarcinoma (STAD), prognostic marker, patient survival, immune infiltration, regulatory T cell, M2 macrophage

## Abstract

Neuropilin1 (NRP1) plays a critical role in tumor progression and immune responses. Although the roles of NRP1 in various tumors have been investigated, the clinical relevance of NRP1 expression in stomach adenocarcinoma (STAD) has not been studied. To investigate the use of NRP1 as a prognostic biomarker of STAD, we analyzed *NRP1* mRNA expression and its correlation with patient survival and immune cell infiltration using various databases. *NRP1* mRNA expression was significantly higher in STAD than normal tissues, and Kaplan-Meier survival analysis showed that *NRP1* expression was significantly associated with poor prognosis in patients with STAD. To elucidate the related mechanism, we analyzed the correlation between *NRP1* expression and immune cell infiltration level. In particular, the infiltration of immune-suppressive cells, such as regulatory T (Treg) cells and M2 macrophage, was significantly increased by *NRP1* expression. In addition, the expression of *interleukin (IL)-35*, *IL-10*, and *TGF-β1* was also positively correlated with *NRP1* expression, resulting in the immune suppression. Collectively in this study, our integrated analysis using various clinical databases shows that the significant correlation between *NRP1* expression and the infiltration of Treg cells and M2 macrophage explains poor prognosis mechanism in STAD, suggesting the clinical relevance of *NRP1* expression as a prognostic biomarker for STAD patients.

## 1. Introduction

Stomach adenocarcinoma (STAD) is one of the most common malignancies and is third in cancer-related deaths worldwide [1]. In addition, its poor prognosis is associated with high invasion, metastasis, and a low early prognosis rate [2]. Recently, advanced surgery and chemotherapy have significantly reduced mortality, however, overall prognosis has not significantly improved [3]. Therefore, the finding of novel biomarkers that allow the prediction of progression and prognosis in patients with STAD have important implications in the development of new drug targets [4,5].

Neuropilin1 (NRP1) is a cell surface glycoprotein involved in various cellular processes, including angiogenesis, cell migration, and axon growth [6]. Especially in various types of tumor, NRP1 has a critical role in cell migration, invasion and angiogenesis, promoting tumor progression [7]. In addition, expression of NRP1 is correlated with poor prognosis in various types of tumor, including lung cancer, colon cancer, ovarian cancer, and prostate cancer [8], implying that NRP1 could be a potential molecular target for treatments of tumor. In gastric cancer, NRP1 also acts as a receptor for major signaling pathways including vascular endothelial growth factor (VEGF), epidermal growth factor (EGF), and semaphorin-3A (SEMA3), showing an important role in tumor development and metastasis [9,10]. NRP1 activates tumor angiogenesis through interaction with VEGF and its receptor and promotes the growth and metastasis of gastric cancer [11,12,13]. Depletion of NRP1 reduces cell proliferation via inhibiting the G1-S phase of cell cycle in gastric cancer cells [11]. Although previous studies have investigated the roles of NRP1 in the progression of gastric cancer in vivo and in vitro, the clinical relevance of NRP1 in gastric cancer has not been fully understood. Therefore, in this study, we performed a comprehensive analysis using various databases and web tools to elucidate NRP1 expression and its correlation with the patient’s clinical outcome in STAD.

In the tumor microenvironment (TME), tumor cells interact with various cells, including immune cells, fibroblasts, and stromal cells [14]. The interaction provides an important environment for tumor immune escape, resulting in the development of various malignant tumors. Particularly, the most abundant immune cells in the TME are known as macrophages [15]. It is well known that macrophages are polarized into two subsets, M1 and M2, which produce a variety of cytokines, proteases and growth factors, acting on the regulation of tumor immunity [16]. M1 macrophages produce type 1 helper T (Th1) cytokines (IL-6, IL-8, IL-12, and tumor necrosis factor (TNF)-α), leading to primarily anti-cancer responses. However, M2 macrophages produce Th2 cytokines such as IL-4, IL-10 and IL-13 to stimulate Th2 immune responses and regulatory T (Treg) cell activation. In the TME, M2 macrophages promote the proliferation of Th2 cells, and induce immune tolerance by activation of Treg cells [17,18,19]. Additionally, it has been reported that M2 macrophages correlate with poor prognosis in gastric cancer. Especially, gastric cancer-derived mesenchymal stromal cells stimulate macrophages to induce M2 macrophage polarization in TME, and the M2 macrophages enhance tumor metastasis, resulting in the poor prognosis of gastric cancer [20]. In addition to M2 macrophages, Treg cells play a critical role in the development and progression of various tumors by their immune-suppressive functions in the TME. A number of studies have shown that a high level of Treg cells acts on innate immune cells and effector T cells to suppress immune responses through secretion of inhibitory cytokines, such as IL-10, TGF-β, and IL-35. In addition, high levels of Treg cells have been observed in patients with various tumors. These are associated with poor prognosis of various types of tumor including gastric cancer, breast cancer and ovarian cancer [19,21,22,23]. Therefore, these studies provide strong evidences that M2 macrophages and Treg cells are highly correlated with tumor immunity and patient prognosis. Moreover, recent studies suggest that the positive feedback between Treg cells and M2 macrophages in TME play an important role in pro-cancer responses and patient prognosis [24,25]. In nasopharyngeal carcinoma (NPC), cancer cells promote M2 macrophage polarization via the release of TGF-β1 and IL-10, and the tumor-infiltrating M2 macrophages induce the recruitment of Treg cells by chemotaxis, resulting in the induction of the density of Treg cells in TME [25].

Interestingly, it has been reported that NRP1 expression increases infiltration of Treg cells in the TME. In vivo study also shows that gene deletion of *NRP1* in tumor infiltrating macrophages exerts an anti-cancer function through suppression of an immune suppression mechanism, and is associated with a better prognosis [25,26]. Therefore, in this study, we investigated *NRP1* mRNA expression and its correlation with prognosis of cancer patients using various databases. As shown in the results, *NRP1* mRNA expression was significantly higher in STAD, compared with normal tissues. The higher expression of *NRP1* was associated with poor patient survival in STAD. Furthermore, *NRP1* expression showed positive correlation with tumor infiltration of Treg cells and M2 macrophages. Collectively, our study suggests that *NRP1* expression could act as an effective prognostic marker by predicting the infiltration of Treg cells and M2 macrophages, indicating the role of *NRP1* as a prognosis biomarker in patients with STAD.

## 2. Experimental Section

### 2.1. Analysis of NRP1 Expression in Various Types of Tumors and Normal Tissues

*NRP1* expression in various cancers and normal tissues was analyzed using the Oncomine, Gene Expression Profiling Analysis (GEPIA2) and Tumor Immune Estimation Resource (TIMER) databases. In the Oncomine database, a tumor microarray database, was used to compare the transcription levels of *NRP1* between tumor and corresponding normal tissues in different types of cancer [27,28]. The threshold was determined according to the following values: p-value < 1 × 10^−4^, fold-change > 2, and gene ranking top 5%. GEPIA2 can assess the effect of 9736 tumors and 8587 normal samples from The Cancer Genome Atlas (TCGA) and the GTEx projects [29,30]. Expression level of *NRP1* across 33 TCGA tumors was compared to normal TCGA and GTEx data using GEPIA2. TIMER database supplies an analysis of relative expression of the gene across tumor and normal tissues [31,32]. *NRP1* expression was analyzed in cancers to compare with normal tissues.

### 2.2. Evaluation of the Relationship between NRP1 Expression and Promoter Methylation in Clinical Characteristics

UALCAN database, using TCGA transcriptome and clinical patient data, provides the expression level of genes and patient characteristics [33,34]. The association between mRNA levels and promoter methylation of *NRP1* and clinicopathological features was analyzed to determine the prognostic value of *NRP1* in patients with stomach adenocarcinoma (STAD). mRNA levels and promoter methylation of *NRP1* were separately analyzed with STAD patient characteristics, including individual cancer stage, age, histological subtype, race, gender, and tumor grade, compared to the normal tissues.

### 2.3. Evaluation of the Relationship between NRP1 Expression and Patient Survival with Various Tumors

The correlation between *NRP1* expression and survival in various cancers was assessed by the GEPIA2 and Kaplan-Meier survival plotter [35]. We used GEPIA to perform overall survival analysis and assessment of the *NRP1* expression levels in STAD and lung adenocarcinoma (LUAD) of the TCGA database. *NRP1* high and low patient groups were split by median NRP1 expression. We assessed cancer prognosis, including overall survival (OS), first progression (FS), and post progression survival (PPS) using gene chip datasets of Kaplan-Meier survival plotter with best cut off option, which split patient groups at the NRP1 expression level to minimize log rank P-value [36]. These data provide the hazard ratio (HR) value with 95% confidence intervals and log-rank *P*-values [29].

### 2.4. Evaluation of the Correlation between NRP1 Expression and Immune Cell Infiltration

We analyzed the correlation between immune cell and *NRP1* expression in STAD using the TIMER database. The correlation between *NRP1* expression and genetic markers of tumor-infiltrating immune cells was explored through the correlation module [31]. The correlation module generated expression scatter plots between a pair of user-defined genes in a given cancer type, along with the Spearman’s correlation and the estimated statistical significance. *NRP1* was used for the *x*-axis with gene symbols, and related marker genes of tumor-associated macrophages (TAM), M1 macrophages, M2 macrophages, and Treg cells were represented on the *y*-axis as gene symbols. Gene expression was displayed with log2 RSEM. Correlation of immune signatures and *NRP1* expression was also confirmed in Tumor Gastric- Tan-192-fRMA-u133p2 dataset in R2: Genomics Analysis and Visualization platform [37].

## 3. Results

### 3.1. mRNA Expression Levels of NRP1 in Various Types of Human Cancer

**Figure 1:**
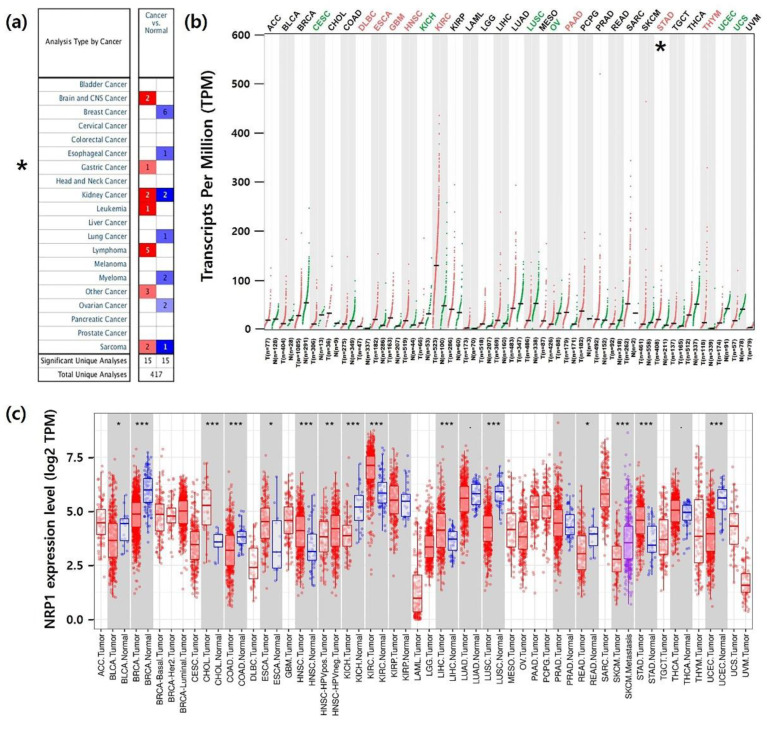
*NPR1* expression levels in different types of cancer and their normal tissues. (**a**) Expression of *NRP1* in different type of cancer using the Oncomine database. The graph shows the numbers of datasets with mRNA up-regulated expression (red) or down-regulated expression (blue). The threshold was designed with following parameters: *p*-value of 1 × 10^−4^ and fold change of 2. (**b**) The GEPIA2 database was used to determine *NRP1* mRNA expression in various types of cancer. The dot plots present mRNA expression data from each cancer and normal tissues that were profiled. Red dots represent tumor tissues, green dots represent normal tissues, and black dots represent the average value of all tumor and normal tissues. The red label indicates that NRP1 is upregulated in cancer compared to normal tissue and green indicates downregulation. The black label indicates no significant difference between the tumor and normal tissue. Abbreviations of cancer types are presented in Supplementary Table S1. (**c**) Expression of *NRP1* in various types of cancer and normal tissues is displayed using the TIMER website. Datasets of normal and cancer tissues were obtained from the TCGA database. * *P* < 0.05, ** *P* < 0.01, *** *P* < 0.001.

To analyze *NRP1* mRNA expression between tumors and normal tissues, we identified *NRP1* mRNA levels using three independent bioinformatics databases. In the Oncomine database, *NRP1* mRNA expression demonstrated upregulation of *NRP1* in lymphoma, brain and central nervous system (CNS), kidney, leukemia, sarcoma, and gastric cancer tissues compared to normal tissues (*p*-value: 1 × 10^−4^, fold-change: 2) (Figure 1a). As shown in Figure 1b, compared with normal tissues, *NRP1* transcript levels were significantly lower in CESC (cervical squamous cell carcinoma and endocervical adenocarcinoma), KICH (kidney chromophobe), LUSC (lung squamous cell carcinoma), OV (ovarian serous cystadenocarcinoma), UCEC (uterine corpus endometrial carcinoma), and UCS (uterine carcinosarcoma). However, it was significantly increased in STAD (stomach adenocarcinoma), DLBC (lymphoid neoplasm diffuse large B-cell lymphoma), ESCA (esophageal carcinoma), GBM (glioblastoma multiforme), HNSC (head and neck squamous cell carcinoma), KIRC (kidney renal clear cell carcinoma), PAAD (pancreatic adenocarcinoma), and THYM (thymoma). In addition, we further analyzed the *NRP1* expression among 36 types of cancer and normal tissues from TCGA data. Figure 1c shows the upregulation of the *NRP1* transcript levels in CHOL (cholangio carcinoma), ESCA (esophageal carcinoma), HNSC (head and neck squamous cell carcinoma), KIRC, and LIHC (liver hepatocellular carcinoma), including STAD. However, downregulation of *NRP1* transcription level was confirmed in BRCA (breast invasive carcinoma), COAD (colon adenocarcinoma), KICH, LUSC, READ (rectum adenocarcinoma), and UCEC. Collectively, data from all three databases shows that *NRP1* mRNA expression in STAD was significantly higher compared to in normal tissues.

### 3.2. Association between NRP1 Expression and Clinical Characteristics in STAD Patients

**Figure 2:**
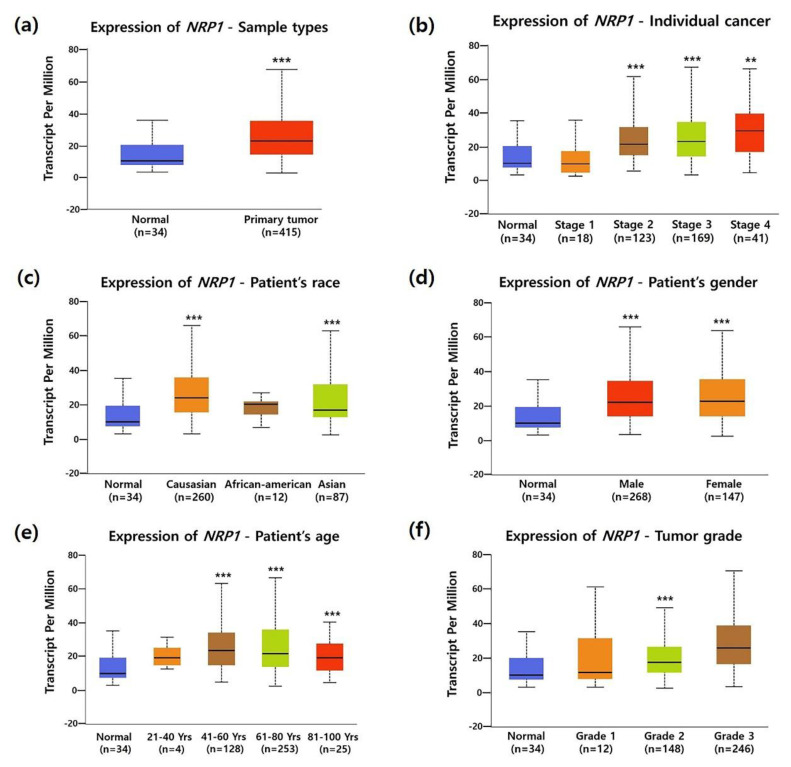
Correlation between *NRP1* expression and clinicopathologic parameters of patients with stomach adenocarcinoma (STAD): *NRP1* mRNA expression level was expressed as box plots using the UALCAN database. Boxplots of mRNA expression of *NRP1* in normal control and STAD tumors with the clinicopathologic parameters such as: (**a**) primary tumors, (**b**) individual cancer stage (how large the primary tumor is and how far the cancer has spread in the patient’s body), (**c**) patient race, (**d**) patient gender, (**e**) patient age, and (**f**) tumor grade (how abnormal the cancer cells look under microscope), were shown. *P* values were results of student’s *t*-test between normal control and each clinicopathologic parameters. * *P* < 0.05, ** *P* < 0.01, *** *P* < 0.001.

**Figure 3:**
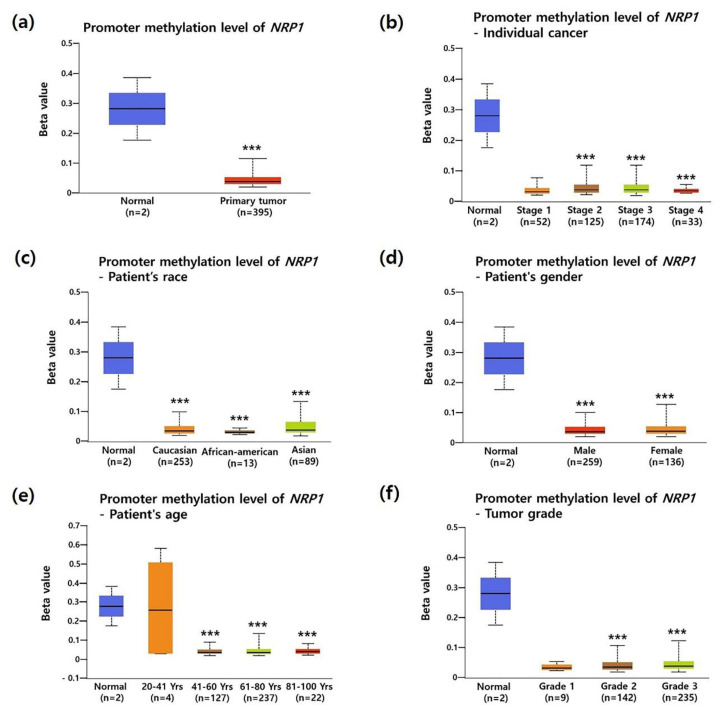
Correlation between promoter methylation of the *NRP1* and clinical parameters of patients with STAD. Promoter methylation level was expressed as box plots using the UALCAN database. Promoter methylation of *NRP1* in STAD tumors (different color plot) and their normal tissues (blue plot) was shown according to the clinicopathologic parameters: (**a**) normal vs. primary tumor, (**b**) individual cancer stage, (**c**) patient race, (**d**) patient gender, (**e**) patient age, and (**f**) tumor grade. * *P* < 0.05, ** *P* < 0.01, *** *P* < 0.001.

To investigate the association between *NRP1* mRNA expression and the clinicopathological characteristics of STAD, we analyzed TCGA data using the UALCAN database. Compared to normal tissues, *NRP1* mRNA expression was significantly increased in most clinicopathological characteristics of STAD; tumor stage (Stage 2, Stage 3 and Stage 4), race (Caucasian and Asian), gender (male and female), age (41–60, 61–80, and 81–100 years old), and tumor grade (Grade 2) as shown in Figure 2a–f. Overexpression of NRP1 mRNA in STAD was further confirmed by another two datasets from Oncomine analysis (Supplementary Figure S2). It is known that promotor methylation is an epigenetic regulatory factor and abnormal methylation is evident in tumors [38]. DNA hypomethylation is found to be involved in the invasion and metastasis processes of gastric cancer cells [39]. In particular, hypomethylated *NRP1* gene has a positive correlation with poor prognosis in gastric cancer [40]. Here, Figure 3a shows that promoter methylation was significantly reduced in STAD compared to normal tissues. Moreover, promotor methylation levels were significantly reduced regardless of patient characteristics, including tumor stage, gender, race, age, and tumor grade (Figure 3b–f). Taken together, these data confirmed upregulation of *NRP1* mRNA expression and downregulation of promoter methylation in STAD.

### 3.3. Association between NRP1 Expression and Prognosis in Patients with STAD

**Figure 4:**
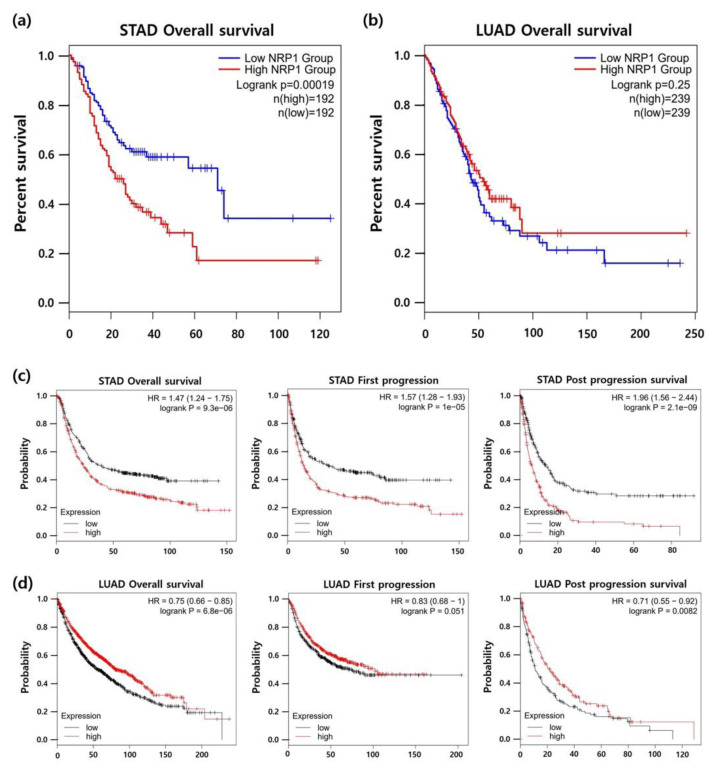
Correlation between *NRP1* expression and patient survival in STAD and lung adenocarcinoma (LUAD). Kaplan-Meier survival curves were generated using the GEPIA2 website. Patient’s survivals were compared between two groups divided at median value of *NRP1* expression as higher (red) and lower (blue) in TCGA data. (**a**) STAD; n = 384, (**b**) LUAD; n = 478. Survival curves of overall survival (OS), first progression (FS), and post progression survival (PPS) in (**c**) STAD and (**d**) LUAD using Kaplan-Meier plotter with best cut-off option [35]. The prognostic value of NRP1 using TIMER database is presented in Supplementary Figure S3.

To elucidate the prognostic value of *NRP1* expression in STAD, we analyzed the correlation between *NRP1* expression and patient survival in STAD using the Kaplan-Meier survival curve. As shown in Figure 4a, high *NRP1* expression was significantly correlated with poor prognosis in STAD. To further confirm the correlation between *NRP1* expression and patient survival, the Kaplan-Meier plotter database was used. Survival curves of overall survival (OS), first progression (FP), and post progression survival (PPS) showed that patient survival rates were considerably decreased by *NRP1* expression (Figure 4c). Interestingly, Figure 4b,d show that there was no association or positive correlation between *NRP1* expression and patient survival in lung adenocarcinoma (LUAD) with no difference in expression of *NRP1* between tumor tissues and normal tissues as shown in Figure 1b,c. Collectively, *NRP1* expression was increased in patients with STAD and had a negative correlation with patient survival in STAD, suggesting that poor prognosis could be predicted through the levels of *NRP1* expression.

### 3.4. Correlation of NRP1 Expression with Treg Cells and M2 Macrophages in STAD

**Figure 5:**
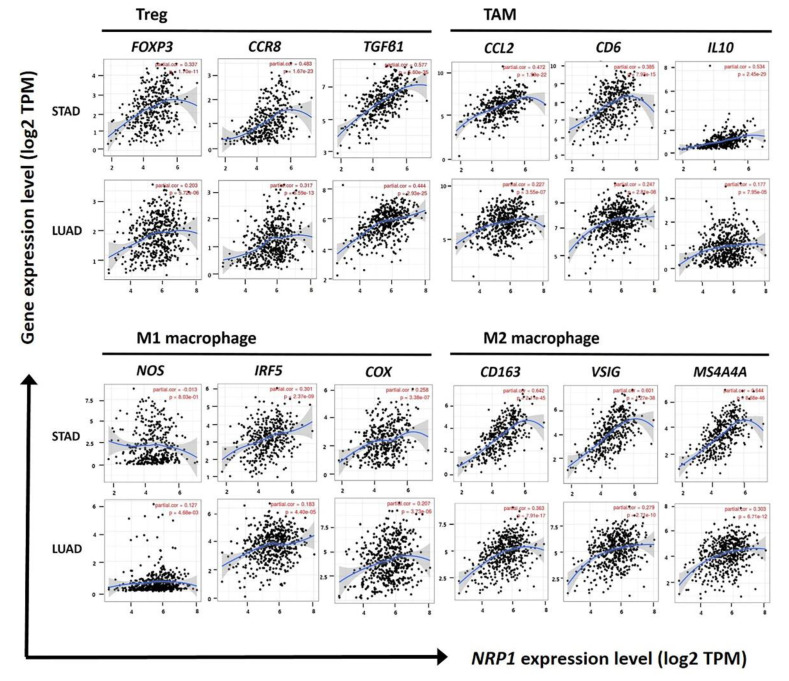
Correlations between *NRP1* expression and gene markers of Treg, TAM, and M1 and M2 macrophages in STAD and LUAD. Infiltration level of *NRP1* expression with various gene markers of Treg cells, TAM, M1 macrophages, and M2 macrophages was confirmed using TIMER. *FOXP3*, *CCR8*, and *TGFβ1* were used as markers for Treg cells, and *CCL2*, *CD68*, and *IL10* were used as markers for TAM. Moreover, *NOS2*, *IRF5*, and *COX2* were used as markers for M1 macrophages, and *CD163, VSIG4*, and *MS4A4A* were used as markers for M2 macrophages. *NRP1* expression was positively correlated with the expression of various gene markers for Treg cells, TAM, and M2 macrophages in STAD. However, the correlation of *NRP1* expression with various gene markers in LUAD was weaker than in STAD. Correlation constants and *P*-values are listed in Table 1.

Tumor microenvironment (TME) consists of immune cells, stromal cells, and soluble factors, as well as cancer cells. It is known that TME plays an important role in the progression of tumors by interacting with other cells [41,42]. Especially, Treg cells and M2 macrophages are well known as potent immunosuppressive cells. Therefore, in TME, Treg cells and M2 macrophages induce the metastasis and growth of tumor cells by inhibiting the anti-tumor function of various effector cells, such as NK cells, CD8^+^ T cells, and γδ T cells [21,43,44]. To investigate factors related to patient survival rates that are regulated by *NRP1* expression, association of *NRP1* expression and infiltration of immune cells was analyzed in STAD. As shown in Figure 5 and Table 1, the correlation between *NRP1* expression and various gene expressions of immune cell markers was determined using TIMER web tool. Most of the immune cell marker genes showed a positive correlation with *NRP1* expression, but among them the marker gene expression of Treg cells and M2 macrophages showed the most strong positive correlations with *NRP1* expression in STAD. Strong positve correlation was confirmed in different dataset using R2 platform as shown in supplementary Table S4. However, there was weak or no significant correlation in LUAD used as negative control compared to STAD.

In immune cells that are generally known to have anti-cancer effects, such as NK cells, CD8^+^ T cells, and B cells, *NRP1* expression also had a weak or moderate positive correlation with their marker gene expression. Additionally, despite the positive correlation between *NRP1* expression and the infiltration of effector cells, higher *NRP1* expression was associated with lower patient survival in STAD, indicating that the infilrated Treg cells and M2 macrophages might effectively inhibit the anti-cancer functions of effector cells. The anti-cancer effects of M1 macrophage and the pro-cancer effects of M2 macrophages and tumor-associated macrophages (TAM) [45,46] are also well known. Interestingly, our data showed that the positive correlation between *NRP1* expression and the infiltration level of TAM and M2 macrophages (*CD163*; cor. = 0.642, *P* = 2.11 × 10^−45^, *VSIG4*; cor. = 0.601, *P* = 1.27 × 10^−38^, *MS4A4A*; cor. = 0.644, *P* = 8.68 × 10^−46^ for M2 macrophages, *CCL2*; cor. = 0.472, *P* = 1.99 × 10^−22^, *CD68*; cor. = 0.384, *P* = 7.92 × 10^−15^, *IL10*; cor. = 0.534, *P* = 2.45 × 10^−29^ for TAM) was much greater than the correlation between *NRP1* expression and the infiltration of M1 macrophages (*NOS2*; cor. = −0.013, *P* = 0.080, *IRF5*; cor. = 0.301, *P* = 2.37 × 10^−9^, *PTGS2*; cor. = 0.258, *P* = 3.38 × 10^−7^). In addition, high correlation between *NRP1* expression and both Treg cells and M2 macrophages was confirmed using the GEPIA database (Table 2). These results suggest that *NRP1* expression significantly affects the infiltration of Treg cells and M2 macrophages, and the infiltrated Treg cells and M2 macrophages enhance tumor progression in STAD by immune-suppressive functions, leading to the poor prognosis.

### 3.5. Correlation between NRP1 Expression and Gene Expression of Immune-Suppressive Cytokines

**Figure 6:**
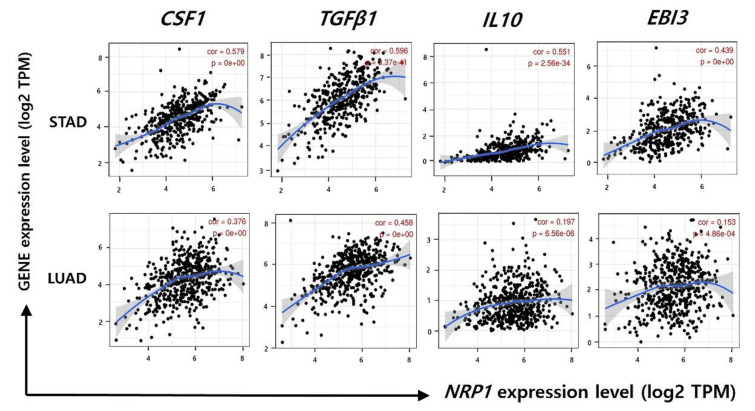
Correlation of *NRP1* expression and gene markers of cytokine (CSF1, TGFβ1, IL-10, and EBI3). Correlation of *NRP1* expression with cytokine gene markers was examined using TIMER. A significant positive correlation between *NRP1* expression and *CSF1*, *TGFβ1*, *IL-10*, and *EBI3* were observed in STAD. However, correlation of NRP1 expression with cytokine gene markers in LUAD was weak compared to STAD. Correlation constants between *NRP1* and gene markers of cytokine are presented in Supplementary Table S5.

Immunosuppression is a well-known mechanism for tumor progression, leading to tumor growth and metastasis. Many studies have reported that Treg cells, M2 macrophages, and TAM contribute to suppress effector cells by various mechanisms, and one of these mechanisms is a secretion of inhibitory cytokines, such as transforming growth factor (TGF)-β1, interleukin (IL)-10, and IL-35 [45,47]. These inhibitory cytokines show anti-cancer effects by immune-suppressive functions on effector cells including NK cell and CD8^+^ T cells [48,49]. In this study, a strong positive correlation was showed between *NRP1* expression and gene expression of Treg cell, M2 macrophage, and TAM markers, indicating the increased infiltration of the immune-suppressive cells (Figure 5, Table 1 and Table 2). Therefore, to investigate whether the immune-suppressive cell-derived cytokines are also increased by *NRP1* expression in STAD, we analyzed the correlation between *NRP1* expression and cytokine gene markers (*CSF1*, *TGFβ1*, *IL10*, *EBI3*) using the TIMER database (Figure 6). Colony-stimulating factor 1 (CSF1) regulates proliferation and differentiation macrophages, resulting in the increased immune-suppressive M2 macrophages [50]. As shown in Figure 6, *NRP1* expression was significantly correlated with the gene expression of immune-suppressive cytokine in STAD (*CSF1*; cor. = 0.551, *P* = 1.79 × 10^−31^, *TGFβ1*; cor. = 0.557, *P* = 5.60 × 10^−35^, *IL10*; cor. = 0.534, *P* = 2.48 × 10^−29^, *EBI3*; cor. = 0.422, *P* = 7.83 × 10^−18^). However, *NRP1* expression was less correlated with the gene expression of immune-suppressive cytokine in LUAD compared to STAD (*CSF1*; cor. = 0.353, *P* = 6.43 × 10^−16^, *TGFβ1*; cor. = 0.444, *P* = 2.93 × 10^−25^, *IL10*; cor. = 0.177, *P* = 7.95× 10^−5^, *EBI3*; cor. = 0.114, *P* = 1.12 × 10^−2^). Collectively, our data indicate that *NRP1* expression had a positive correlation with immune-suppressive functions by up-regulating inhibitory cytokines, resulting in the poor prognosis in STAD.

## 4. Discussion

Neuropilin1 (NRP1) acts as a co-receptor of various growth factors and plays a major role in tumor progression. Overexpression of NRP1 is associated with poor prognosis in various cancers including prostate cancer, lung cancer, and melanoma. However, it has been reported to be associated with favorable prognosis in colon cancer [51,52,53]. The contradictory prognostic value of NRP1 expression among types of cancers might be caused by the tumor-specific roles of NRP1 and its related mechanisms. This study demonstrates that high expression of *NRP1* correlates with poor prognosis of stomach adenocarcinoma (STAD). In addition, the expression of *NRP1* was highly correlated to the invasion of various immune cells. Particularly there was a clear difference in correlation of *NRP1* expression with infiltrated Treg and M2 macrophages between STAD and lung adenocarcinoma (LUAD). Therefore, our study suggests differential TME between STAD and LUAD that affects prognostic value of *NRP1* expression. Increased *NRP1* mRNA and protein expression in stomach cancer tissue was already suggested in a previous report [11]. We confirmed higher expression of *NRP1* mRNA in stomach cancer at multiple datasets using the various databases (Oncomine, GEPIA2, TIMER, and UALCAN) (Figure 1 and Figure 2). In addition, the results using the UALCAN database indicate that *NRP1* mRNA expression was significantly increased in tumor tissues compared to in normal tissues, regardless of clinical characteristics, such as tumor stage, age, race, gender, and tumor grade of STAD using TCGA dataset (Figure 2).

Interestingly, the promoter methylation of *NRP1* exhibited a significant decrease across almost all clinical characteristics. DNA methylation mainly regulates the level of gene expression. Especially, abnormal methylation is frequently observed in tumors by affecting the cell division cycle, therefore it is associated with tumor malignant biological properties [54]. The cytosine-phosphate-guanine (CpG) island methylator phenotype (CIMP), which exhibits extensive methylation, has been associated with lymph node metastasis in gastric cancer [55]. In particular, *NRP1* methylation, among the tumor-related genes, is found to be associated with survival of colon and liver cancers [53,56]. In addition, *NRP1* is a hypomethylated and upregulated gene in tumor tissues and is co-expressed with platelet-derived growth factor receptor beta (PDGFRB), which is associated with the malignant phenotype for patients with gastric cancer, thereby potentially serving as a prognostic biomarker of gastric cancer [40]. Here, our data also showed significantly higher expression of *NRP1* and lower DNA methylation of *NRP1* gene in STAD than in normal control. Taken together, it suggests that the hypomethylation of *NRP1* could be associated with poor prognosis of STAD.

Next, to suggest the possibility of *NRP1* as a marker to predict the prognosis of STAD, we analyzed the correlation between *NRP1* expression and patient survival using the Kaplan-Meire curve. *NRP1* expression, as based on the Kaplan-Meier survival curves, was significantly correlated with overall survival (OS), first progression (FP), and post progression survival (PPS) in STAD, showing that *NRP1* expression is negatively correlated with patient survival. (Figure 4). NRP1 has been suggested as a therapeutic target for cancers owing to its tumor promoting roles in various cancers [57,58,59,60]. In gastric cancer, NRP1 expression confers cancer stemness with binding to Lin28B [61]. NRP1 induced a JNK-dependent signaling cascade leading to resistance to BRAF, HER2, or MET inhibitors [62]. Knockdown of NRP1 expression by miR-338 inhibited gastric cancer cell migration, invasion, proliferation and promoted apoptosis [63]. NRP1 depletion inhibited cell proliferation and migration by inhibiting multiple receptors in which NRP1 plays a role as coreceptor [11]. However, the value of NRP1 expression in the tumor immune microenvironment in stomach cancer remains to be studied although NRP1 is regarded as an immune-related gene and its expression is negatively correlated with patient OS [64].

This study also demonstrates that *NRP1* expression correlates with immune cell infiltration in stomach cancer. As shown in Figure 5 and Table 1, a strong correlation between *NRP1* in STAD and various immune cells was confirmed. Interestingly, infiltration levels of effector T cells, CD8^+^ T cells, NK cells, and B cells, moderately correlated with *NRP1* expression compared to Treg cells and M2 macrophages (Table 1). Despite a slight correlation with infiltration level of effector cells, the strong correlation of *NRP1* expression with infiltration level of Treg cells and M2 macrophages suggested the possible relation of *NRP1* expression to immune suppression in STAD. Tumor associated macrophages (TAM), which present properties of the M2 macrophages in the tumor microenvironment (TEM), are known to induce immune suppression and stimulate tumor progression [65,66]. The common action of M2 macrophages and TAM is presumed to be a major cause of the similar data for the M2 macrophages and TAM in this study. High levels of infiltration of Treg cells and M2 macrophages in the immune environment correlate with the patient prognosis in other type of cancer [67,68]. Treg cells and M2 macrophage have immune suppressive mechanisms including inhibition of antigen presenting cell (APC) maturation and secretion of inhibitory cytokines [68,69,70,71]. Activated Treg cells also evade tumor immunity by inhibiting effector cells, such as CD4^+^ and CD8^+^ T cells [72]. In addition, tumor infiltrated M2 macrophages exhibit a tumorigenic effect by producing high levels of IL-10 and TGF-β1 [73]. NRP1 enhances Treg cells for tumor infiltration and promotes macrophage differentiation, as preparation for the activation of immune evasion responses [60,74]. Therefore, this study suggests that *NRP1* expression induces the production of the inhibitory cytokines IL-10, IL-35, and TGFβ1 of the Treg cells and M2 macrophages, and the expressed cytokines serve as major signals in the immune suppression mechanism of STAD.

Tumor infiltrated Treg cells and M2 macrophages significantly correlate with the tumor suppressive system in STAD as they produce cytokines such as transforming growth factor beta 1 (TGF-β1), interleukin-10 (IL-10), and colony stimulating factor 1 (CSF1) [16]. CSF1, which regulates the macrophage lineage, has been found to increase the expression of macrophages and TAM, control the differentiation and function of M2 macrophages, and act as an angiogenesis switch [71,75,76]. According to a related study, the gene inhibition of CSF1 in breast cancer cells affects tumor formation in immunodeficient mice [73]. In addition, multiple tumor cell injections in the mouse liver metastasis models were found to increase the number of CD4+ CD25 + Treg cells and the expression of cytokines IL-10 and TGFβ-1 [77]. IL-35, which inhibits the inflammatory response of immune cells, is a dimeric protein with IL-12A and Epstein-Barr Virus-induced 3 (EBI3), which are encoded by IL-12A and EBI3 individual genes [78,79]. The present study confirmed that *NRP1* expression correlated with cytokine gene markers (*CSF1*, *TGFβ1*, *IL10*, *EBI3*) that were secreted from Treg cells and M2 macrophages (Figure 6). Therefore, the expression of *NRP1* affects the cytokines produced by Treg cells and M2 macrophage and indicates poor prognosis for patients with STAD by the immune suppression mechanism. Since this study has demonstrated the clinical significance of *NRP1*, in vitro and in vivo studies are needed to further demonstrate the role of *NRP1* in immunosuppression mechanisms.

## 5. Conclusions

The main finding of this study is that *NRP1* expression is positively correlated in Treg cells and M2 macrophages in STAD. Further, we found a significant correlation between *NRP1* expression and cytokines in Treg cells and M2 macrophages. High correlation of *NRP1* expression with immunosuppressive cells may be one of the causes of the prognostic value of NRP1 in STAD. Thus, this systematic analysis provides evidence suggesting the potential use of *NRP1* as an effective biomarker for patient survival in STAD and a therapeutic target modulating the tumor immune microenvironment.

## Figures and Tables

**Table 1:** Correlation analysis between NRP1 and gene markers of immune cells using TIMER.

Description	Gene Markers	STAD				LUAD			
		None		Purity		None		Purity	
		Cor	*P*	Cor	*P*	Cor	*P*	Cor	*P*
**T cell (general)**	*CD3D*	0.306	***	0.273	***	0.143	*	0.101	0.024
	*CD3E*	0.333	***	0.39	***	0.155	**	0.117	*
	*CD2*	0.382	***	0.36	***	0.168	**	0.127	*
**Treg cell**	*FOXP3*	0.363	***	0.336	***	0.233	***	0.203	***
	*CCR8*	0.495	***	0.482	***	0.331	***	0.317	***
	*STAT5B*	0.579	0	0.574	***	0.364	***	0.358	***
	*TGFβ (TGFB1)*	0.596	***	0.576	***	0.458	0	0.444	***
**TAM**	*CCL2*	0.501	0	0.472	***	0.247	***	0.227	***
	*CD68*	0.414	0	0.384	***	0.256	***	0.247	**
	*IL10*	0.551	***	0.534	***	0.197	***	0.177	*
**M1 Macrophage**	*INOS(NOS2)*	−0.001	0.976	−0.013	0.803	0.151	**	0.127	*
	*IRF5*	0.313	***	0.301	***	0.206	***	0.183	***
	*COX2 (PTGS2)*	0.279	***	0.258	***	0.192	***	0.207	**
**M2 Macrophage**	*CD163*	0.662	0	0.642	***	0.365	***	0.363	***
	*VSIG4*	0.607	0	0.601	***	0.290	***	0.279	***
	*MS4A4A*	0.656	0	0.644	***	0.309	***	0.303	***
**CD8^+^ T cell**	*CD8A*	0.365	***	0.339	***	0.156	**	0.129	*
	*CD8B*	0.220	***	0.202	***	0.077	0.081	0.049	0.280
**Neutrophil**	*CD66b(CEACAM8)*	0.102	0.036	0.115	0.025	0.193	***	0.183	***
	*CD11b (ITGAM)*	0.614	0	0.598	***	0.367	***	0.357	***
	*CCR7*	0.427	0	0.403	***	0.157	**	0.119	*
**NK cell**	*KIR2DL1*	0.250	***	0.256	***	0.015	0.725	−0.003	0.939
	*KIR2DL3*	0.180	***	0.169	***	0.089	0.043	0.069	0.123
	*KIR2DL4*	0.113	0.02	0.086	0.091	0.068	0.122	0.044	0.324
	*KIR3DL1*	0.219	***	0.210	***	0.021	0.629	−0.011	0.810
	*KIR3DL2*	0.204	***	0.202	***	0.122	0.005	0.100	0.026
	*KIR3DL3*	−0.001	0.991	0.022	0.667	0.064	0.145	0.042	0.352
	*KIR2DS4*	0.162	***	0.161	*	0.093	0.034	0.065	0.151
**B cell**	*CD19*	0.259	***	0.230	***	−0.008	0.850	−0.057	0.203
	*CD79A*	0.263	***	0.219	***	0.040	0.369	0.004	0.925
**Monocyte**	*CD86*	0.575	***	0.563	***	0.340	***	0.332	***
	*CD115 (CSF1R)*	0.664	0	0.647	***	0.374	***	0.357	***
**T cell exhaustion**	*PD1 (PDCD1)*	0.266	***	0.245	***	0.157	**	0.127	*
	*CTLA4*	0.289	***	0.265	***	0.186	***	0.152	**
	*LAG3*	0.249	***	0.218	***	0.076	0.086	0.043	0.343
	*TIM3 (HAVCR2)*	0.569	0	0.553	***	0.309	***	0.296	***

STAD, Stomach Adenocarcinoma; LUAD, Lung Adenocarcinoma; Treg, regulatory T cell; TAM, tumor-associated macrophage; Th, T helper cell; Tfh, Follicular helper T cell; Cor, *P* value of Spearman’s correlation; None, correlation without adjustment; Purity, correlation adjusted by purity. * *P* < 0.01; ** *P* < 0.001; *** *P* < 0.0001.

**Table 2:** Correlation analysis between *NRP1* expression and genes markers of Treg cells, TAM, M1 macrophages, and M2 macrophages using GEPIA 2.

Cell Type	Gene Markers	STAD				LUAD			
		Tumor		Normal		Tumor		Normal	
		*R*	*P*	*R*	*P*	*R*	*P*	*R*	*P*
**Treg**	*FOXP3*	0.35	***	−0.36	0.03	0.21	***	0.31	0.019
	*CCR8*	0.5	***	−0.22	0.19	0.33	***	0.25	0.059
	*STAT5B*	0.62	***	0.87	***	0.37	***	0.84	***
	*TGF* *β (TGFB1)*	0.59	***	0.17	0.33	0.43	***	0.43	**
**TAM**	*CCL2*	0.48	***	0.41	0.013	0.23	***	0.12	0.36
	*CD68*	0.46	***	−0.32	0.057	0.29	***	0.11	0.42
	*IL10*	0.58	***	0.2	0.25	0.21	***	0.024	0.86
**M1 Macrophage**	*INOS (NOS2)*	0.017	0.74	0.28	0.1	0.19	***	0.62	***
	*IRF5*	0.59	***	0.67	***	0.29	***	−0.19	0.15
	*COX2 (PTGS2)*	0.33	***	0.77	***	0.2	***	0.21	0.11
**M2 Macrophage**	*CD163*	0.59	***	0.67	***	0.29	***	−0.19	0.15
	*VSIG4*	0.6	***	0.64	***	0.28	***	−0.2	0.12
	*MS4A4A*	0.64	***	0.61	***	0.31	***	−0.22	0.099

** *P* < 0.001; *** *P* < 0.0001.

## Supplementary Materials

The following are available online at https://www.mdpi.com/2077-0383/9/5/1430/s1. Supplementary Table S1; Tumor Abbreviations, Supplementary Figure S2; Overexpression of NRP1 mRNA in STAD from further Oncomine Analysis, Supplementary Figure S3. The prognostic value of NRP1 using TIMER database. Supplementary Table S4; Correlation analysis between NRP1 and gene markers of immune cells using R2 database, Supplementary Table S5; Correlation constants and *p*-values in Figure 6.
