# Supplementary material for: Neuropilin1 Expression Acts as a Prognostic Marker in Stomach Adenocarcinoma by Predicting the Infiltration of Treg Cells and M2 Macrophages
Source: J Clin Med. 2020 May 12;9(5):1430. doi: 10.3390/jcm9051430 (PMC7290937; doi:10.3390/jcm9051430)
Supplement: Supplementary file 1 [file jcm-09-01430-s001.pdf]

Supplementary Table S1. Tumor Abbreviations.

Abbreviation	Type of Cancer
ACC	Adrenocortical carcinoma
BLCA	Bladder Urothelial Carcinoma
BRCA	Breast invasive carcinoma
CESC	Cervical squamous cell carcinoma and endocervical adenocarcinoma
CHOL	Cholangio carcinoma
COAD	Colon adenocarcinoma
DLBC	Lymphoid Neoplasm Diffuse Large B-cell Lymphoma
ESCA	Esophageal carcinoma
GBM	Glioblastoma multiforme
HNSC	Head and Neck squamous cell carcinoma
KICH	Kidney Chromophobe
KIRC	Kidney renal clear cell carcinoma
KIRP	Kidney renal papillary cell carcinoma
LAML	Acute Myeloid Leukemia
LGG	Brain Lower Grade Glioma
LIHC	Liver hepatocellular carcinoma
LUAD	Lung squamous cell carcinoma
MESO	Mesothelioma
OV	Ovarian serous cystadenocarcinoma
PAAD	Pancreatic adenocarcinoma
PCPG	Pheochromocytoma and Paraganglioma
PRAD	Prostate adenocarcinoma
READ	Rectum adenocarcinoma
SARC	Sarcoma
SKCM	Skin Cutaneous Melanoma
STAD	Stomach adenocarcinoma
TGCT	Testicular Germ Cell Tumors
THCA	Thyroid carcinoma
THYM	Thymoma
UCEC	Uterine Corpus Endometrial Carcinoma
UCS	Uterine Carcinosarcoma
UVM	Uveal Melanoma

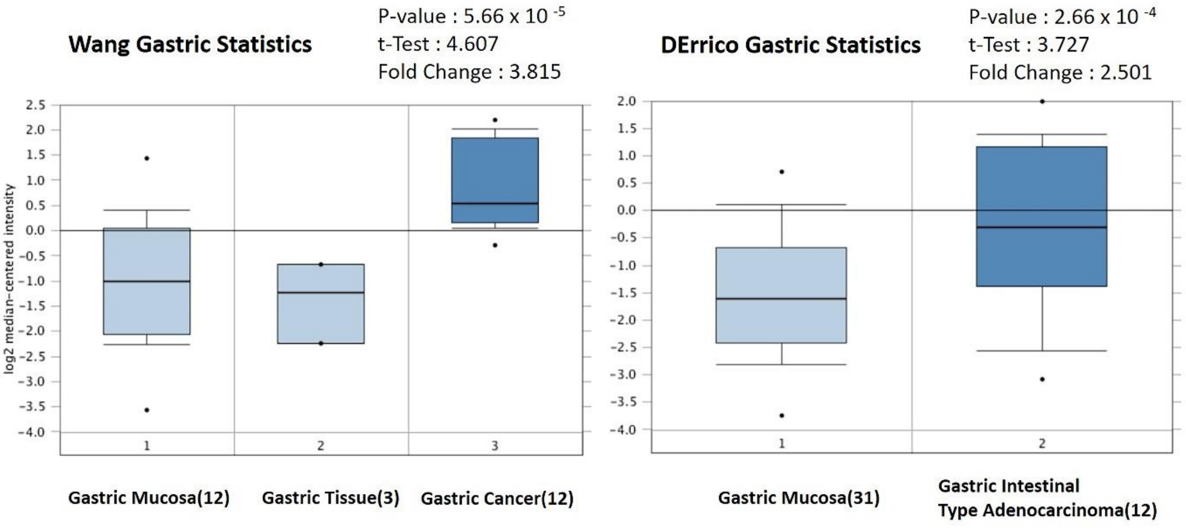

Supplementary Figure S2. Overexpression of NRP1 mRNA in STAD from further Oncomine Analysis.

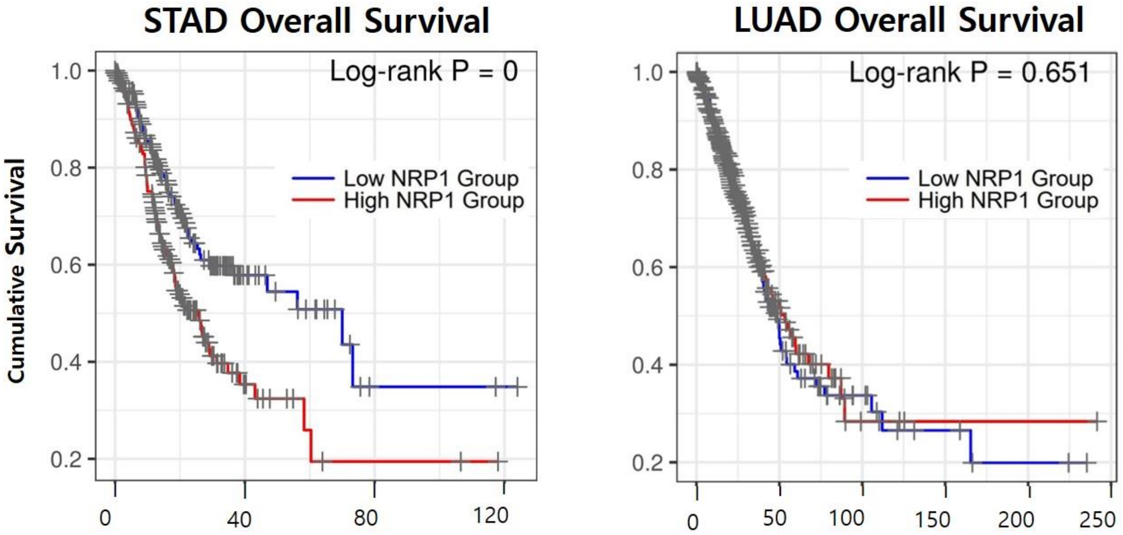

Supplementary Figure S3. The prognostic value of NRP1 using TIMER database.

Supplementary Table S4. Correlation analysis between NRP1 and gene markers of immune cells using Tumor-Gastric-Tan-192-fRMA-u133p2 dataset in R2: Genomics Analysis and Visualization platform (http://r2.amc.nl).

**Description**	**Gene markers**	**STAD**	
		**r**	***P***
	CD3D	0.032	0.656
**T cell (general)**	*CD3E*	0.234	***
	CD2	0.239	***
	FOXP3	-0.296	***
**Treg cell**	*CCR8*	-0.147	0.042
	STAT5B	0.637	***
	TGFβ (TGFB1)	0.689	***
	CCL2	0.722	***
**TAM**	*CD68*	0.163	0.024
	IL10	0.356	***
	INOS(NOS2)	-0.326	***
**M1 Macrophage**	*IRF5*	-0.161	0.026
	COX2 (PTGS2)	0.395	***
	CD163	0.717	***
**M2 Macrophage**	*VSIG4*	0.691	***
	MS4A4A	0.727	***
**CD8⁺ T cell**	*CD8A*	0.129	0.075
	CD8B	0.004	0.957
**Neutrophil**	*CD66b(CEACAM8)*	-0.025	0.729
	CD11b (ITGAM)	0.570	***
	CCR7	0.058	0.421
**NK cell**	*KIR2DL1*	-0.474	***
	KIR2DL3	-0.480	***
	KIR2DL4	-0.396	***
	KIR3DL1	-0.350	***
	KIR3DL2	-0.350	***
	KIR3DL3	-0.230	***
	KIR2DS4	-0.344	***
**B cell**	*CD19*	-0.184	0.011
	CD79A	-0.145	0.044

**Monocyte**	*CD86*	0.564	***
	CD115 (CSF1R)	0.612	***
	PD1 (PDCD1)	-0.426	***
**T cell exhaustion**	*CTLA4*	-0.082	0.260*
	LAG3	-0.001	1.000
	TIM3 (HAVCR2)	0.515	***

Supplementary Table S5. Correlation constants and p-values in Figure 6.

**Gene markers**	**STAD**				**LUAD**			
	**None**		**Purity**		**None**		**Purity**	
	**Cor**	***P***	**Cor**	***P***	**Cor**	***P***	**Cor**	***P***
CSF1	0.579	0 x 10^{-00}	0.551	1.79 x 10^{-31}	0.376	0 x 10^{-00}	0.353	6.43 x 10^{-16}
TGFβ1 (TGFB1)	0.596	3.37 x 10^{-41}	0.577	5.60 x 10^{-35}	0.458	0 x 10^{-00}	0.444	2.93 x 10^{-25}
IL10	0.551	2.56 x 10^{-34}	0.534	2.48 x 10^{-29}	0.197	6.56X10^{-6}	0.177	7.95 x 10^{-5}
EBI3	0.43	0 x 10^{-00}	0.422	7.83 x 10^{-18}	0.153	4.86 x 10^{-4}	0.114	1.12 x 10^{-2}
